# The Proteome of Neuromelanin Granules in Dementia with Lewy Bodies

**DOI:** 10.3390/cells11223538

**Published:** 2022-11-09

**Authors:** Maximilian Wulf, Katalin Barkovits, Karin Schork, Martin Eisenacher, Peter Riederer, Manfred Gerlach, Britta Eggers, Katrin Marcus

**Affiliations:** 1 Medizinisches Proteom-Center, Medical Faculty, Ruhr-University Bochum, 44801 Bochum, Germany; 2 Medical Proteome Analysis, Center for Proteindiagnostics (PRODI), Ruhr-University Bochum, 44801 Bochum, Germany; 3 Center of Mental Health, Department of Psychiatry, Psychosomatics and Psychotherapy, University Hospital of Wuerzburg, 97080 Wuerzburg, Germany; 4 Psychiatry Department of Clinical Research, University of Southern Denmark Odense University Hospital, 5000 Odense, Denmark; 5 Center of Mental Health, Department of Child and Adolescent Psychiatry, Psychosomatics and Psychotherapy, University Hospital of Wuerzburg, University of Wuerzburg, 97070 Wuerzburg, Germany

**Keywords:** neuromelanin granules, *substantia nigra pars compacta*, neurodegeneration, dementia with Lewy bodies, proteomics, stress granules

## Abstract

Neuromelanin granules (NMGs) are organelle-like structures present in the human *substantia nigra pars compacta*. In addition to neuromelanin, NMGs contain proteins, lipids and metals. As NMG-containing dopaminergic neurons are preferentially lost in Parkinson’s disease and dementia with Lewy bodies (DLB), it is assumed that NMGs may play a role in neurodegenerative processes. Until now, this role is not completely understood and needs further investigation. We therefore set up an exploratory proteomic study to identify differences in the proteomic profile of NMGs from DLB patients (n = 5) compared to healthy controls (CTRL, n = 5). We applied a laser microdissection and mass-spectrometry-based approach, in which we used targeted mass spectrometric experiments for validation. In NMG-surrounding (SN_Surr._) tissue of DLB patients, we found evidence for ongoing oxidative damage and an impairment of protein degradation. As a potentially disease-related mechanism, we found α-synuclein and protein S100A9 to be enriched in NMGs of DLB cases, while the abundance of several ribosomal proteins was significantly decreased. As S100A9 is known to be able to enhance the formation of toxic α-synuclein fibrils, this finding points towards an involvement of NMGs in pathogenesis, however the exact role of NMGs as either neuroprotective or neurotoxic needs to be further investigated. Nevertheless, our study provides evidence for an impairment of protein degradation, ongoing oxidative damage and accumulation of potentially neurotoxic protein aggregates to be central mechanisms of neurodegeneration in DLB.

## 1. Introduction

Dementia with Lewy bodies (DLB) is an age-related, neurodegenerative disease, which is affecting approximately 0.1% of the population above 65 [1]. Until now, the pathogenesis remains partially unknown, even though there is evidence for several contributing pathological mechanisms, such as oxidative stress or mitochondrial dysfunction [2,3,4,5,6]. Still, there are no curative treatment options available. One major hallmark of DLB defines the loss of dopaminergic neurons in the *substantia nigra* (SN) *pars compacta*, visible by a loss of pigmentation [7,8]. This pigmentation is a result of the deposition of neuromelanin, a black-brownish pigment, stored in so-called neuromelanin granules (NMGs), located in the cell bodies of dopaminergic neurons. The mechanisms behind the generation of neuromelanin, as well as origin and function of NMGs remain elusive [9,10].

Since NMGs are absent in common laboratory animals [11], such as mice or rats, researchers rely on *post-mortem* brain tissue. Proteomic studies on SN tissue and enriched NMGs from cases without history of neurological diseases (CTRL) regularly reported a link between NMGs and lysosomes, while a recent study from our group provided hints for a potential relation between NMGs and stress granules, eventually indicating a role of NMGs in protein synthesis or a potential mechanism of their generation [12,13,14,15,16].

The role of NMGs in synucleinopathies especially needs further investigation [17,18,19,20,21], as NMG-containing neurons of the SN are preferentially lost in Parkinson’s disease (PD) and DLB, which both share several pathological mechanisms but are clinically separated based on the occurrence of Parkinsonism and dementia as well as co-pathologies [7,8,22]. The preferential loss of pigmented neurons was further supported for other NMG-containing areas in the midbrain, in which the grade of pigmentation correlated positively with the proportion of neuronal degeneration [7,23,24,25,26,27,28], meaning that areas containing high numbers of pigmented neurons are affected most by neuronal loss in PD. Although these findings assume an involvement of NMGs in the pathogenesis in neurodegenerative diseases such as DLB, molecular characterization of NMGs from DLB in comparison to NMGs from CTRL cases is missing until now. Only two comparable studies were conducted so far, of which one compared transcriptome and proteome levels of dopaminergic neurons in the SN of PD and CTRL cases [29]. While the researchers found several PD-related genes and proteins to be dysregulated, they reported the overall correlation between transcriptome and proteome level to be low. The other study conducted proteome analyses of *post-mortem* brain tissue showing Lewy body pathology without the focus on a specific disease, which for example revealed RNA-binding proteins to play a role in Lewy body formation [30].

Our previously published paper focusing on the differences in the proteomic profile of NMGs and SN tissue surrounding NMGs (SN_Surr._) provided us with numerous novel insights concerning the biological role of NMGs [16]. As one of our key findings, we were able to provide evidence for ribosomal, RNA-binding and translation-associated proteins to be highly abundant in NMGs, which may indicate a link between NMGs and stress granules, thus implying NMGs to be somehow involved in protein synthesis. In addition, we could show that tyrosine hydroxylase (TH) and aromatic-L-aminoacid decarboxylase (DDC), proteins required for dopamine synthesis, are significantly higher abundant in NMGs than in SN_Surr._ tissue, pointing towards a potential production of dopamine in NMGs.

Based on these promising results, we transferred our established strategy to tissue of DLB cases. We provide evidence of impaired mechanisms for protein degradation as well as ongoing oxidative stress in SN_Surr._ tissue of DLB cases, the results of which are in line with regularly reported pathomechanisms in DLB. In addition, we found α-synuclein to be highly abundant in NMGs of DLB patients, which we could verify by targeted mass spectrometry, hinting towards a potential involvement of NMGs in the progression of neurodegeneration in DLB due to the eventual formation of neurotoxic α-synuclein fibrils.

## 2. Materials and Methods

### 2.1. Disclosure

The presented data concerning the proteome of NMGs from CTRL cases was discussed in detail in another publication and is reused in this study [16]. Still, mass spectrometric experiments of samples obtained from CTRL and DLB cases were performed at the same time to ensure comparability. Therefore, experimental procedures are similar to the previously published study.

### 2.2. Ethical Statement

Five µm thick slices of human *post-mortem* SN tissue were provided on 1.0 PEN-membrane glass slides (Carl Zeiss Microscopy GmbH, Göttingen, Germany) by the Navarrabiomed Biobank (Pamplona, Spain). The use of human brain tissue was approved by the ethics committee of the Ruhr-University Bochum, Germany (file number 4760-13), according to German regulations and guidelines. Ethical approval was obtained at 12 November 2013.

### 2.3. Subjects

Fresh frozen SN tissue slices of five CTRL cases were used as well as tissue slices of five patients *post-mortem* diagnosed with DLB based on common international criteria [31]. Based on pathological measures, DLB cases were classified as Braak stages 4–6 [32]. Additional information can be found in Table 1. Additionally, SN tissue slices of two CTRL and DLB cases independent of the study cohort as well as slices of two cases of both conditions included in the study cohort were used for validation of differential protein abundances by parallel reaction monitoring (PRM)-experiments. Since the sample size is quite limited, this study has to be seen as a first exploration of the proteome of NMGs in DLB cases.

### 2.4. Laser Microdissection

Tissue slices were stored at −80 °C until further use. NMGs and SN_Surr._ tissue were isolated as displayed in Supplementary Figure S1 and previously reported [16,33]. In a first step, NMGs corresponding to a tissue area of 500,000 µm^2^ were isolated at 400×-magnification and collected in water-filled caps of non-adhesive microtubes (MicroTube 500, Carl Zeiss Microscopy GmbH, Jena, Germany) using a PALM Micro Beam (P.A.L.M.-System, Carl Zeiss Microscopy GmbH). After that, 1,000,000 µm^2^ of SN_Surr._ tissue was selected at 50×-magnification in the same tissue areas from which NMGs were isolated in the first step, and collected in a fresh tube. All samples were stored at −80 °C until further use. In addition, tissue not previously depleted of NMG was excised and collected in separate tubes. These samples were thereafter treated in the same way as the NMG samples, but were excluded from data analysis using Perseus.

### 2.5. Sample Preparation

The sample preparation was performed as previously reported [16,33]. At first, samples were thawed, briefly centrifuged and dried in a vacuum-centrifuge (Concentrator plus, Eppendorf AG, Hamburg, Germany). Afterwards, samples are refilled with formic acid (FA) (VWR International GmbH, Darmstadt, Germany) and incubated in FA for 20 min at room temperature. After incubation, samples were sonicated in an ice-cooled sonication bath (USC300TH, VWR International GmbH, Darmstadt, Germany) for five minutes and vacuum-dried. After that, samples were refilled with ammonium bicarbonate (Sigma-Aldrich Chemie GmbH, Taufkirchen, Germany), reduced with dithiothreitol for 30 min at 56 °C (AppliChem GmbH, Darmstadt, Germany), and acetylated with iodoacetamide (Merck KGaA, Darmstadt, Germany) at room temperature in the dark. Trypsin (SERVA Electrophoresis GmBH, Heidelberg, Germany), diluted in ammonium bicarbonate to 0.1 µg/µL per 1,000,000 µm^2^ tissue, was then added to the samples according to the collected tissue area and digestion was performed at 37 °C overnight. The digestion was stopped after ~16 h by adding trifluoroacetic acid (Merck KGaA, Darmstadt, Germany). Next, samples were vacuum-dried, and peptides were stored in 20 µL of 0.1% TFA at −80 °C.

### 2.6. Mass Spectrometric Analysis

To ensure comparability of mass spectrometric measurements, the settings remained as previously reported [16]. Peptide samples were diluted in 0.1% TFA for liquid-chromatography tandem mass spectrometry (LC-MS/MS) analysis. During this step, the volume of peptide solution was adapted according to the amount of collected tissue for NMG and SN_Surr._ samples to ensure equal sample load. Mixtures of all samples were measured over the course of all LC-MS/MS measurements to check for technical variances. An Ultimate 3000 RSLC nano LC system (Dionex, Idstein, Germany) coupled to an Orbitrap Fusion Lumos Tribrid mass spectrometer (Thermo Fisher Scientific, Waltham, MA, USA) was used for LC-MS/MS measurements. Peptides were loaded on a pre-column (Acclaim PepMap nanoViper, Thermo Fisher Scientific, Waltham, MA, USA; 100 µm × 2 cm, 5 µm particle size) and washed with 0.1% TFA at a flow rate of 30 µL/min for 7 min. Afterwards, the pre-column was connected to an analytical C18 column (Acclaim PepMap nanoViper, Thermo Fisher Scientific, Waltham, USA; 75 µm × 50 cm, 2 µm particle size). Separation of peptides was performed at a flow rate of 400 nL/min with a gradient starting with 95% solution A (0.1% FA) and 5% solution B (84% acetonitrile, 0.1% FA). The concentration of solution B was increased up to 30% after 105 min, then within 2 min to 95% and maintained at that level for 3 further minutes. Subsequently, the column was adjusted back to 5% solution B. These gradient settings were used for data dependent acquisition (DDA)-experiments, as well as for PRM-experiments.

#### 2.6.1. Global Mass Spectrometry

For global DDA-experiments, the system operated within a scan range from 350 to 1400 m/z (mass-to-charge) with a resolution of 120,000 and a maximum injection time of 80 ms. In a fixed cycle time of 2 s, all precursor ions with an intensity above 1 × 10^4^ were selected for fragmentation at a fixed collision energy of 28% by higher-energy collisional dissociation (HCD). Precursor ions selected for fragmentation were maintained on a dynamic exclusion list for 30 s. Fragment ion scans were performed at a resolution of 30,000 with a maximum injection time of 80 ms.

#### 2.6.2. Targeted Mass Spectrometry

For PRM-experiments, the system operated within a scan range of 350 to 1400 m/z with a resolution of 120,000 and a maximum injection time of 50 ms. An inclusion list containing 12 precursor ions, representing unique peptides of α-synuclein, TH, glycerol-3-phosphate dehydrogenase and cytoplasmic dynein 1 heavy chain 1 was applied (Table 2). For each peptide precursor, a retention time (RT) window of 3 min was defined in the inclusion list. During the RT window, the peptide precursor was selected by the Quadrupole for fragmentation at a collision energy of 28% by HCD. The fragment ion scan was carried out in the Orbitrap at a resolution of 60,000 with a maximum injection time of 200 ms.

The mass spectrometry proteomics data have been deposited to the ProteomeXchange Consortium via the PRIDE partner repository with the dataset identifier PXD035310 [34].

### 2.7. Data Analysis and Statistics

Data analysis and statistical evaluation were performed as previously described to ensure the comparability of results [16].

#### 2.7.1. Global Mass Spectrometry

Raw files were analyzed using the MaxQuant software (version 1.6.17.0, https://www.maxquant.org/, accessed on 17 December 2020). Resulting peak lists were searched against the human UniProt FASTA reference proteome (version 01/20/2021; 75,796 entries) and a common contaminants database provided in the Andromeda search engine [35,36,37]. Carbamidomethlyation of cysteine was set as fixed modification and carbamidomethylation of any N-terminus, oxidation of methionine as well as deamidation of asparagine and glutamine were set as variable modifications due to sample preparation. Trypsin was set as digestion enzyme and a maximum number of 2 missed cleavages were tolerated in the search. The false discovery rate (FDR), determined by searching against a reverse decoy database, was set to 1% for peptides (minimum length of seven amino acids) and proteins. Peptide identification was carried out with an initial allowed precursor mass deviation up to 5 parts per million (ppm) and an allowed fragment mass deviation of 20 ppm. Match between runs was enabled to enhance and optimize peptide identification. Label-free quantification (LFQ) was performed using the MaxQuant LFQ normalization whereby the Top10 unique and razor peptides were assessed for protein quantification [38]. Intensity-based absolute quantification (iBAQ) value calculation was enabled as well.

The resulting protein group output was analyzed using the Perseus software (version 1.6.15.0, https://maxquant.net/perseus/, accessed on 3 February 2021) [39], as part of the MaxQuant environment, whereby master mixes and samples of SN tissue not depleted of NMGs were excluded for the subsequent analysis. Contaminants and decoys were filtered out and LFQ values were subsequently log2 transformed. For further analysis, NMG and SN_Surr._ samples were analyzed separately.

Two main strategies were applied, the first focusing on getting the broadest possible insight into the proteomic profiles represented in our datasets, therefore focusing on normalized mean iBAQ values as measures for protein abundances in each sample group. These values were used for Figures 2 and 4 as well as Tables 3 and 4. IBAQ values were divided by the sum of iBAQ values in that sample as normalization. Of these normalized iBAQ values, mean values were created for each sample group (NMG CTRL, NMG DLB, SN_Surr._ CTRL, SN_Surr._ DLB). Proteins with normalized mean iBAQ values > 0 were considered as identified. For the ranking plots shown in the manuscript, ranked normalized mean iBAQ values were multiplied by 1000 and displayed on a logarithmic scaling. For this strategy, no statistical testing was applicable.

Our second strategy focused on the label-free quantification of proteins based on LFQ values to get reliable insights into the protein quantities present in NMGs and SN_Surr._ tissue of CTRL and DLB cases. A minimum of 70% valid values per group was set as an additional filtering criterion before remaining missing values were imputed from a normal distribution using a width of 0.3 and a downshift of 1.8. Paired Student’s t-test as well as *p*-value correction by Benjamini-Hochberg were applied to the dataset (leading to q-values) to determine proteins with significantly differential abundances. Protein groups (as a summary of proteins which are not differentiable by mass spectrometric measurements are hereafter referred to as proteins) with a *p*-value < 0.05 were defined as being significantly differential, while q-values were applied as more stringent statistical criteria. Fold changes were determined by calculating the ratio of means between the two assessed groups (CTRL and DLB) for NMG and SN_Surr._ samples.

The results of this analysis are presented as two Volcano plots, created in R version 4.0.3 (https://www.r-project.org/, accessed on 4 January 2021) [40] using the packages ggplot2 3.3.3 and ggrepel 0.9.1. Negative log10-transformed *p*-values (uncorrected) are plotted against the log2-transformed fold changes. Proteins that reach a *p*-value < 0.05 are marked in red. On each side, the ten proteins with the largest Euclidean distance from the origin in the Volcano plot are labeled with the corresponding gene name.

For principal component analysis (PCA), analysis was conducted and graphics were created in R using the same software versions as for the Volcano plot. LFQ values from MaxQuant were taken and log2-transformed, zero values were treated as missing values. Proteins with more than 50% missing values were excluded and the remaining missing values were imputed by the mean of the corresponding protein intensities. A PCA was calculated using the setting that scales the data beforehand. The first two principal components (PC) were plotted.

To search for information on specific proteins, the UniProt database was utilized [41].

#### 2.7.2. Targeted Mass Spectrometry

The verification of a selection of differentially expressed proteins with significant *p*-values was carried out by PRM experiments for α-synuclein, TH, glycerol-3-phosphate dehydrogenase and cytoplasmic dynein 1 heavy chain 1. For that, the resulting peptide output of MaxQuant was processed in an identical manner as our protein group output and peptides assigned to TH and α-synuclein were examined for significantly differential expression (*p*-value < 0.05), fold change, retention time and charge state. As housekeeping proteins, glycerol-3-phosphate dehydrogenase and cytoplasmic dynein 1 heavy chain 1 were chosen, as they were found to be neither significant nor differential between NMGs and SN_Surr._ tissue and additionally displayed a low standard deviation on the level of intensities. Evaluation of PRM data was performed using Skyline software (version 20.2, https://skyline.ms/project/home/software/Skyline/begin.view, accessed on 5 February 2021 [42]. Group comparisons were performed on peak areas for each protein and an adjusted *p*-value < 0.05, according to Benjamini-Hochberg, was used as a significance threshold on MS2-level.

## 3. Results

### 3.1. The Proteome of Neuromelanin Granules and Surrounding Tissue Remains Highly Similar under Neurodegenerative Conditions

Since the comparison of proteomic profiles of NMGs and SN_Surr._ tissue of CTRL cases provided us with several novel insights concerning open questions on NMGs, we extended our investigations by analyzing DLB patients to gain a deeper insight into the role of NMG in disease progression and pathogenesis. In total, we identified 2559 proteins (Supplementary Table S1), of which 1246 proteins were found to be suitable for the systematic quantitative comparison.

**Figure 1:**
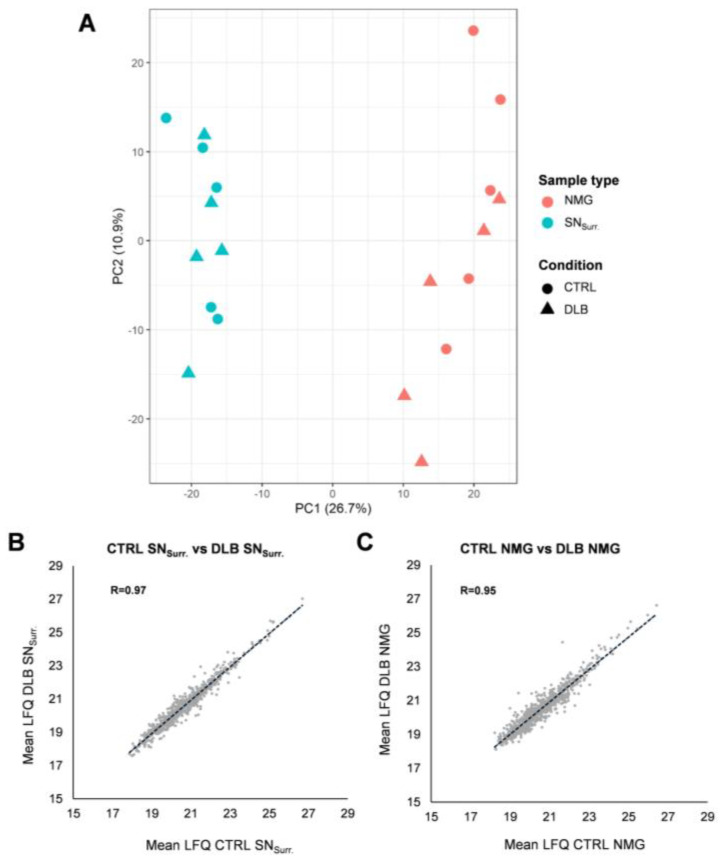
Statistical evaluation of our dataset. (**A**): Principal Component Analysis (PCA) of NMG and SN_Surr._ samples of DLB and CTRL cases shows a high similarity in the proteomic profile of NMGs and SN_Surr._ tissue between both conditions. In contrast, NMG and SN_Surr._ samples are highly separated on Principal Component 1. (**B**): Mean LFQ values for proteins in CTRL and DLB SN_Surr._ samples are highly comparable. The Pearson correlation coefficient of 0.97 supports this even further. (**C**): Comparison of mean LFQ values for proteins in CTRL and DLB NMGs reveals the high similarity between both conditions, which is also stressed by the Pearson correlation coefficient of 0.95.

In a first step, we aimed to assess similarities and differences in the global protein pattern of NMGs and SN_Surr._ tissue in health and disease. Our Principal Component analysis (Figure 1A) showed a clear separation between NMGs and SN_Surr._ tissue (Principal Component 1), thus we can state that NMGs and SN_Surr._ tissue exhibit a sample-type specific protein profile. In addition, we verified that NMGs and SN_Surr._ samples of CTRL and DLB cases are not clearly separated (Principal Component 2), suggesting a high similarity in their proteomic profiles.

To verify this high similarity in protein abundances in either NMGs or SN_Surr._ tissue of CTRL and DLB cases, we compared the abundance (mean LFQ values) for each protein in SN_Surr._ (Figure 1B) or NMG (Figure 1C) samples of DLB and CTRL cases. To substantiate this finding, we further calculated the correlation coefficients of protein intensities, which were found to be very high for both sample types (SN_Surr._; R = 0.97, NMG; R = 0.95), again stressing the proteomic profile of NMGs and SN_Surr._ Samples to be consistent even under neurodegenerative conditions.

### 3.2. Dopaminergic Marker Proteins Are Reduced in NMG-Surrounding Tissue of DLB Cases

The loss of dopaminergic neurons in the SN is a regularly reported hallmark of synucleinopathies like PD and DLB. Therefore, we focused on the comparison of the proteomic profile of SN_Surr._ tissue of DLB and CTRL cases first, as it should provide a general insight into ongoing neuropathological changes in the SN.

In a first step, we wanted to assess the influence of neuropathology in DLB on important cell types of the SN, respectively neurons, dopaminergic neurons, astrocytes and oligodendrocytes. Therefore, we investigated the abundance of proteins being characteristic for each of these cell types (neurons: SNCA, NEFL, NEFH, MAPT, NEFM, APP; dopaminergic neurons: TH, DDC, SLC6A3; astrocytes: GFAP, S100B; oligodendrocytes: MBP, MOG, MAG).

**Figure 2:**
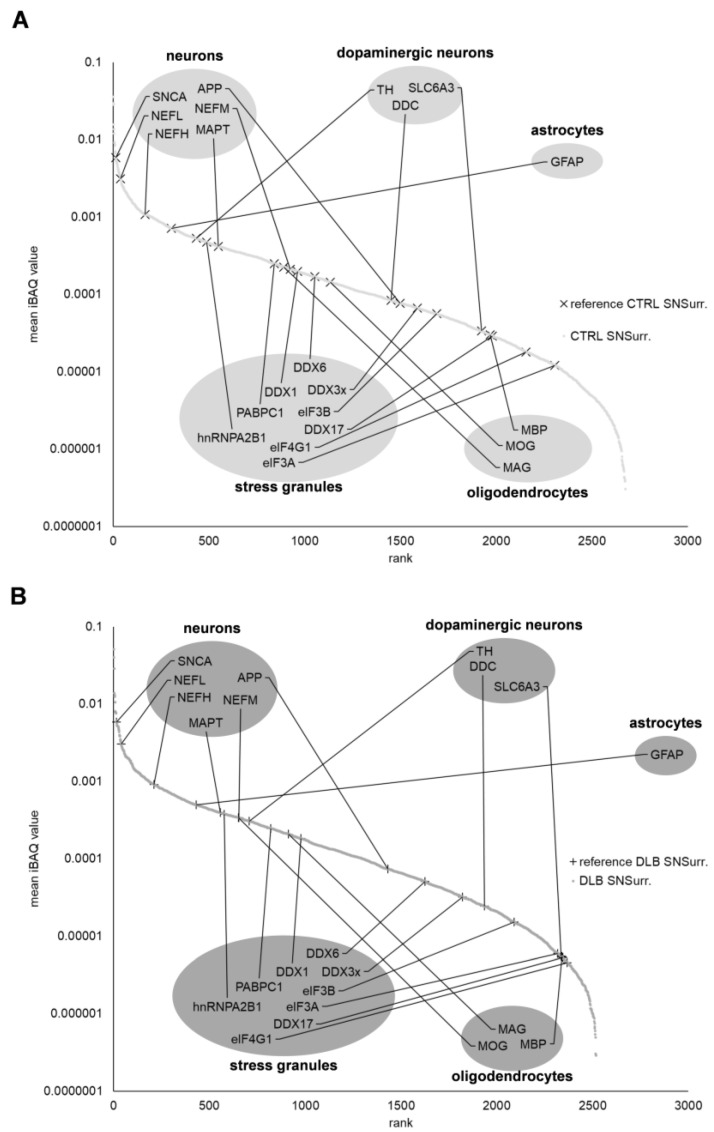
Ranking of protein expression intensities in SN_Surr._ tissue of both conditions. Plots display the normalized mean iBAQ-values of identified proteins in SN_Surr._ tissue of CTRL (**A**) and DLB (**B**) cases. General neuronal marker proteins as well as marker for stress granules, astrocytes and oligodendrocytes are comparable based on their ranks in CTRL and DLB SN_Surr._ tissue, while marker proteins for dopaminergic neurons rank lower in SN_Surr._ tissue of DLB cases. SNCA: α-synuclein, NEFL: neurofilament light chain, NEFM: neurofilament medium chain, NEFH: neurofilament heavy chain, APP: amyloid beta precursor protein, MAPT: microtubule associated protein tau, TH: tyrosine hydroxylase, DDC: aromatic-L-amino-acid decarboxylase, SLC6A3: solute carrier family 6 member 3, MBP: myelin basic protein, GFAP: glial fibrillary acidic protein, MAG: myelin-associated glycoprotein, MOG: myelin-oligodendrocyte glycoprotein, hnRNPA2B1: heterogeneous nuclear ribonucleoproteins A2/B1, PABPC1: polyadenylate-binding protein 1, DDX1: ATP-dependent RNA helicase DDX1, DDX6: probable ATP-dependent RNA helicase DDX6, DDX3x: ATP-dependent RNA helicase DDX3X, eIF3B: eukaryotic translation initiation factor 3 subunit B, eIF3A: eukaryotic translation initiation factor 3 subunit A, DDX17: probable ATP-dependent RNA helicase DDX17, eIF4G1: eukaryotic translation initiation factor 4 gamma 1.

To assess the protein expression intensities of these markers in health and disease, we ranked all identified proteins in regards to their iBAQ values (Supplementary Table S2, Figure 2). In general, both proteomic profiles were highly comparable, as general neuronal marker proteins such as α-synuclein (SNCA), neurofilament light chain (NEFL) and microtubule-associated protein tau (MAPT), were found to be equally expressed in both CTRL and DLB cases. As a marker for astrocytes, only GFAP was identified in our samples with slightly decreased abundance in NMGs of DLB cases. While oligodendrocyte markers (MBP, MOG, MAG) were slightly increased in SN_Surr._ tissue of DLB cases, especially marker proteins for dopaminergic neurons were decreased in their abundance in SN_Surr._ tissue of DLB cases. These results indicate the regularly reported specific decrease of dopaminergic neurons in the SN of DLB cases, while other neuronal marker proteins do not seem to be affected.

In a second step, we analyzed the abundance of stress granule markers (hnRNPA2B1, PABPC1, DDX1, DDX6, DDX3x, eIF3B, DDX17, eIF4G1, eIF3A) in SN_Surr_. tissue, as our recent publication, as well as current literature propose an alternative hypothesis, in which stress granules may be linked to the abnormal aggregation of proteins in neurodegenerative diseases like synucleinopathies. However, five of the nine assessed stress granule marker proteins (DDX3x, DDX6, DDX17, eIF3B, eIF4G1) showed reduced protein expression intensities in SN_Surr._ tissue under neurodegenerative conditions.

### 3.3. Impaired Protein Degradation and Oxidative Stress May Contribute to Neurodegeneration in NMG-Surrounding SN Tissue

**Figure 3:**
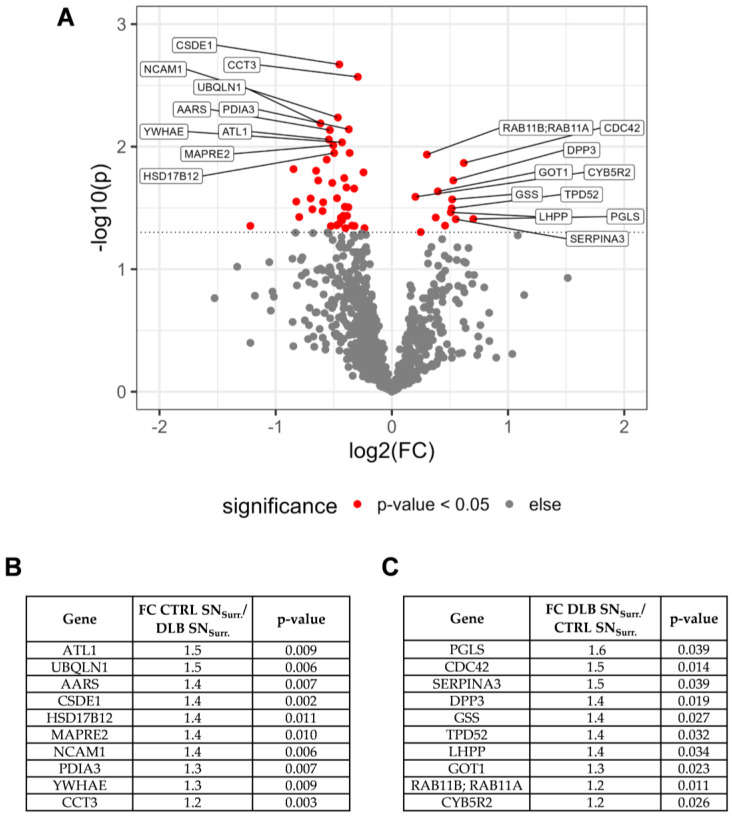
Results of the quantitative comparison of proteins in SN_Surr._ tissue of CTRL and DLB cases. (**A**): Volcano plot displaying −log_10_-transformed *p*-values and log_2_-transformed fold changes (FC) of proteins matching the criteria for the quantitative comparison. Proteins with highest Euclidean distances on both sides are indicated with their respective gene symbols. (**B**): Summary of fold change and *p*-value for proteins with highest Euclidean distances and higher abundances in SN_Surr._ tissue of CTRL cases. (**C**): Summary of fold change and *p*-value for proteins with highest Euclidean distances and higher abundances in SN_Surr._ tissue of DLB cases. ATL1: atlastin-1; UBQLN1: ubiquilin-1; AARS: alanine-tRNA ligase, cytoplasmic; CSDE1: cold shock domain-containing protein E1; HSD17B12: very-long-chain 3-oxoacyl-CoA reductase; MAPRE2: microtubule-associated protein RP/EB family member 2; NCAM1: neural cell adhesion molecule 1; PDIA3: protein disulfide-isomerase A3; YWHAE: 14-3-3 protein epsilon; PGLS: 6-phosphogluconolactonase; CDC42: cell division control protein 42 homolog; SERPINA3: alpha-1-antichymotrypsin; DPP3: dipeptidyl peptidase 3; GSS: glutathione synthetase; TPD52: tumor protein D52; phospholysine phosphohistidine inorganic pyrophosphate phosphatase; GOT1: aspartate aminotransferase, cytoplasmic; RAB11B: ras-related protein Rab-11B; RAB11A: ras-related protein Rab-11A; CYB5R2: NADH-cytochrome b5 reductase 2.

To gain a more detailed view into potential pathomechanisms present in the SN of DLB cases, we aimed to identify proteins being differently expressed between CTRL and DLB SN_Surr_. tissue. Our quantitative comparison led to the identification of 13 proteins being higher abundant in DLB cases, and 41 proteins being higher abundant in SN_Surr._ tissue of CTRL cases (Supplementary Table S1). As especially those proteins with high fold changes and low *p*-values may be relevant to understand the ongoing pathological changes in the SN of DLB patients, we focused on the top ten proteins regarding the combination of both criteria (Figure 3A–C) first.

Among these proteins, glutathione synthetase (GSS) was enriched in SN_Surr._ tissue of DLB cases, which may indicate an upregulation of the production of antioxidative glutathione in SN_Surr._ tissue of DLB cases, potentially as a response to the presence of reactive oxygen species (ROS). On the other hand, as ubiquilin-1 (UBQLN1) was higher abundant in SN_Surr._ tissue of CTRL cases, the ubiquitin-proteasome system (UPS) and thus the capability to degrade accumulated proteins may be decreased in the SN of DLB cases. Surprisingly, considering the frequently reported loss of dopaminergic neurons in the SN of DLB patients, TH levels were not significantly reduced (*p* = 0.17) but showed a clear trend towards a reduction in SN_Surr._ tissue of DLB patients (fold change CTRL/DLB: 2.9).

### 3.4. α-Synuclein Is Highly Abundant in NMGs of DLB Cases

**Figure 4:**
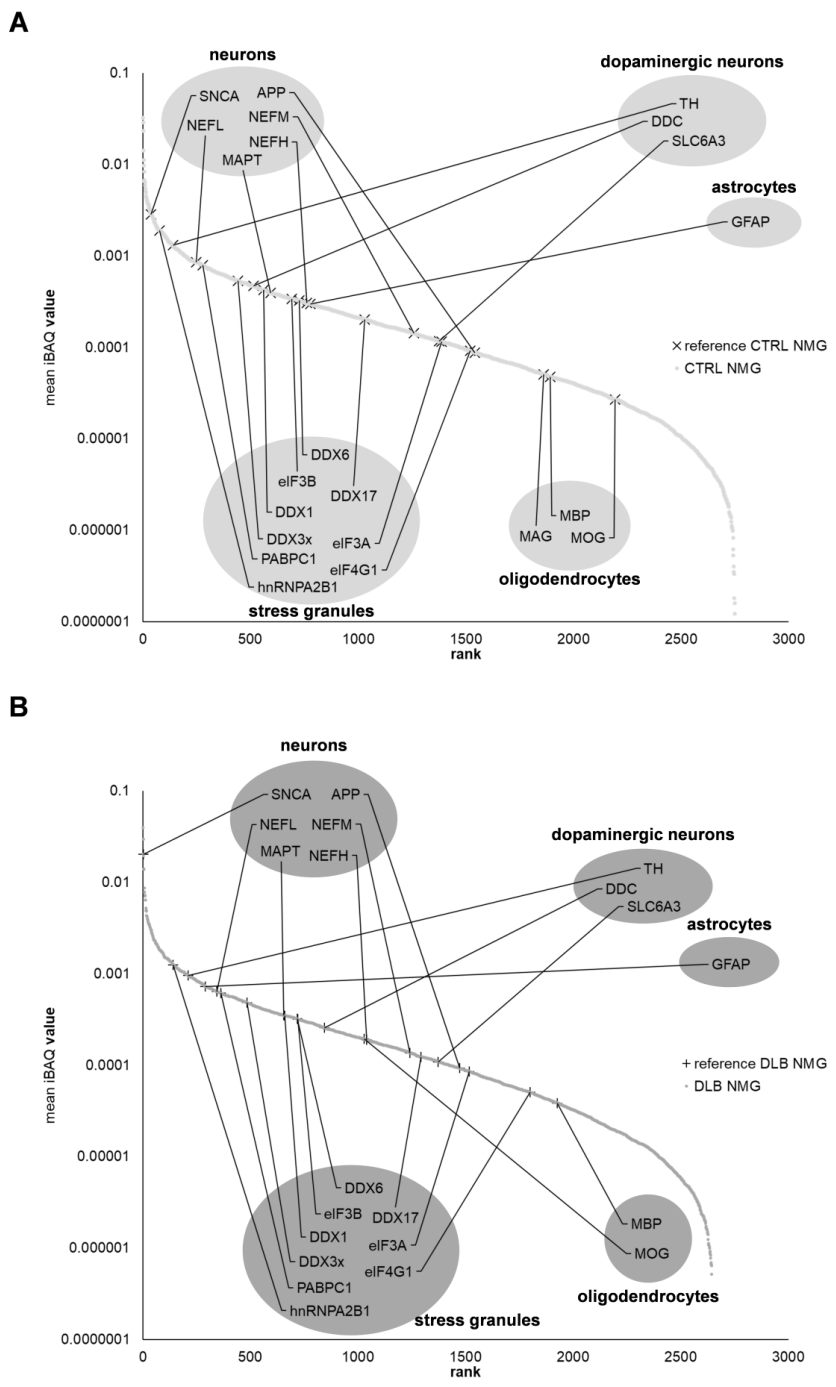
Ranking of protein expression intensities in NMGs of both conditions. Plots display the normalized mean iBAQ-values of identified proteins in NMGs of CTRL (**A**) and DLB (**B**) cases. While α-synuclein is enriched in abundance and amongst the highest abundant proteins in NMGs of DLB cases, dopaminergic marker proteins and some stress granule marker proteins are reduced in abundance, thus higher abundant in CTRL NMGs. GFAP, the only identified marker protein for astrocytes, was found to be higher abundant in NMGs of DLB cases.

Since especially neuromelanin containing dopaminergic neurons are lost in the SN of DLB cases, it is assumed that neuromelanin or NMGs may be involved in the pathogenesis of DLB. Thus, we subsequently focused on potential proteomic changes within NMGs of DLB cases. We first aimed for neuronal marker proteins as well as marker proteins for dopaminergic neurons, astrocytes and oligodendrocytes within CTRL and DLB NMGs (Supplementary Table S2, Figure 4).

As expected, marker proteins for oligodendrocytes were only of low abundance in NMGs of both conditions, while GFAP, the only identified marker protein for astrocytes, was found to be higher abundant in NMGs of DLB cases. General marker proteins for neurons remained comparably abundant in NMGs of DLB and CTRL cases with SNCA being the only exception, as it showed an immense increase in abundance in NMGs of DLB cases. Furthermore, we detected a remarkable decreased abundance of TH and DDC (aromatic-L-amino-acid decarboxylase), two major dopaminergic markers in NMGs of DLB cases, confirming one of the major hallmarks of DLB, the loss of dopaminergic neurons.

Subsequently, we demonstrated an altered distribution of stress granule proteins within NMGs of DLB cases. Five marker proteins for stress granules (DDX1, DDX17, eIF3A, eIF4G1 and PABPC1) were decreased in abundance in DLB cases compared to CTRL cases, while other stress granule markers (DDX3x, DDX6, eIF3B, hnRNPA2B1) remained comparably abundant. In summary, this global view provided us with first insights into an enrichment of SNCA in NMGs of DLB cases, while dopaminergic and stress granule marker proteins were reduced in abundance.

### 3.5. Alpha-Synuclein and Protein S100A9 Are Highly Abundant in NMGs of DLB Cases

**Figure 5:**
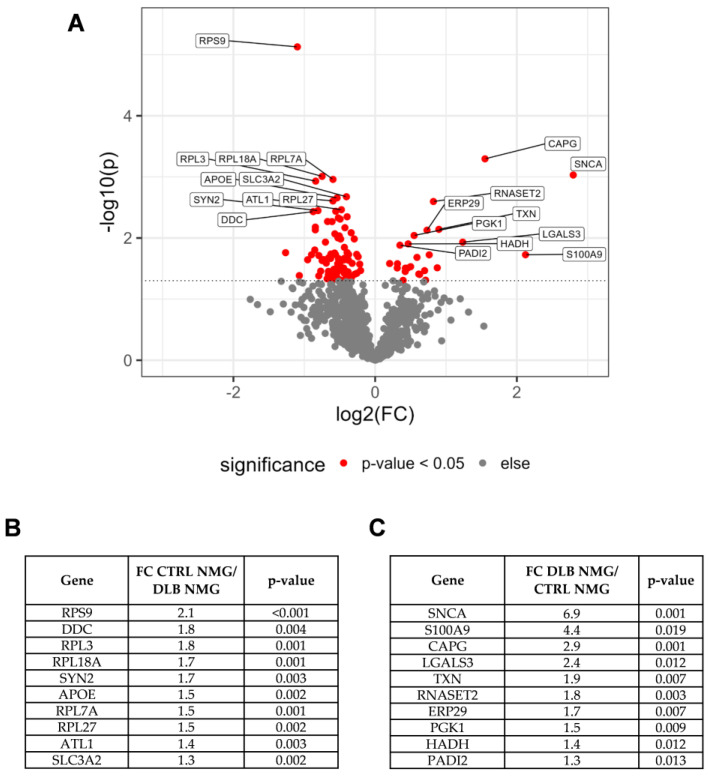
Results of the quantitative comparison of proteins in NMGs of CTRL and DLB cases. (**A**): Volcano plot displaying −log_10_-transformed *p*-values and log_2_-transformed fold changes of proteins matching the criteria for the quantitative comparison. Proteins with highest Euclidean distances on both sides are indicated with their respective gene symbols. (**B**): Summary of fold change and *p*-value for proteins with highest Euclidean distances and higher abundances in NMGs of CTRL cases. (**C**): Summary of fold change and *p*-value for proteins with highest Euclidean distances and higher abundances in NMGs of DLB cases. RPS9: 40S ribosomal protein S9; DDC: aromatic-L-amino-acid decarboxylase; RPL3: 60S ribosomal protein L3; RPL18A: 60S ribosomal protein L18a; SYN2: synapsin-2; APOE: apolipoprotein E; RPL7A: 60S ribosomal protein L7a; RPL27: 60S ribosomal protein L27; ATL1: atlastin-1; SLC3A2: 4F2 cell-surface antigen heavy chain; SNCA: α-synuclein; S100A9: protein S100-A9; CAPG: macrophage-capping protein; LGALS3: galectin-3; TXN: thioredoxin; RNASET2: ribonuclease T2; ERP29: endoplasmic reticulum resident protein 29; PGK1: phosphoglycerate kinase 1; HADH: hydroxyacyl-coenzyme A dehydrogenase, mitochondrial; PADI2: protein-arginine deiminase type-2.

In a next step, we aimed to identify altered protein abundances hinting towards potential pathomechanisms present in NMGs of DLB cases. Our analysis identified 25 proteins to be significantly higher abundant in NMGs of DLB cases, while 85 proteins were higher abundant in NMGs of CTRL cases (Figure 5 and Supplementary Table S1).

We decided to focus on the top ten proteins first. Among the ten proteins with higher abundances in CTRL NMGs (Figure 5A in red, left and Figure 5B), five belonged to either the small or the large ribosomal subunit (RPS9, RPL3, RPL18A, RPL7A, RPL27), indicating both ribosomal subunits to be higher abundant in NMGs of CTRL cases. In addition, both proteins required for dopamine synthesis, TH and DDC, were found to be more highly abundant in NMGs of CTRL cases (Supplementary Table S1). Among the proteins being significantly more abundant in NMGs of DLB cases, α-synuclein and protein S100A9 displayed the highest fold changes with 6.9 and 4.4, respectively (Figure 5C). Since α-synuclein is known to accumulate in characteristic protein aggregates in DLB, this accumulation in NMGs may be a disease-specific effect.

### 3.6. High Abundances of α-Synuclein in NMGs of DLB Cases Can Verified by Targeted Mass Spectrometry

**Figure 6:**
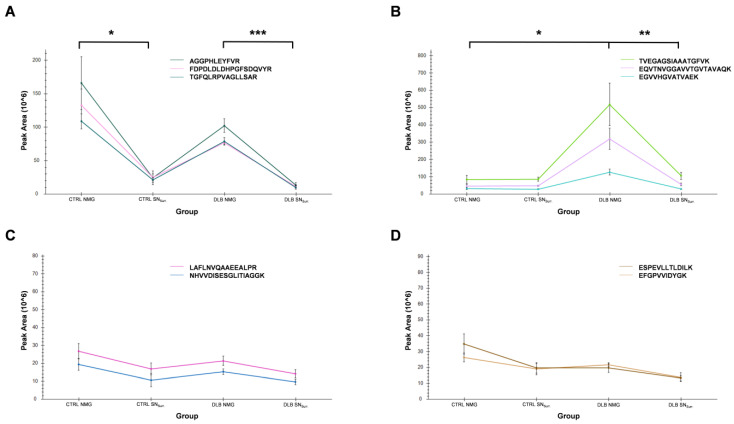
Parallel reaction monitoring (PRM) results for TH (**A**) and α-synuclein (**B**) as well as two reference proteins (**C**, **D**). Graphs show intensities of selected peptides as mean values for each sample group. TH was of significantly higher abundance in NMGs of CTRL and DLB cases, compared to SN_Surr._ tissue. α-synuclein was significantly enriched in DLB NMGs compared to DLB SN_Surr._ tissue and CTRL NMGs. As expected, glycerol-3-phosphate-dehydrogenase and cytoplasmic dynein 1 heavy chain 1 showed comparable intensities in all sample groups, providing evidence for loading of comparable sample amount. *: adjusted *p*-value < 0.05; **: adjusted *p*-value < 0.01; ***: adjusted *p*-value < 0.001.

To validate our results, we established a targeted mass spectrometric method to elucidate total protein abundances in our samples (Figure 6). For that, we chose α-synuclein and TH due to their high scientific interest regarding NMG origin and function. We additionally selected two proteins which were found to be similarly abundant among all samples, glycerol-3-phosphate-dehydrogenase and cytoplasmic dynein 1 heavy chain 1, as a control. As expected, glycerol-3-phosphate-dehydrogenase (Figure 6C) and cytoplasmic dynein 1 heavy chain 1 (Figure 6D) showed comparable abundances in all four sample groups and no significant differences.

With our targeted experiments, we could further confirm that TH was significantly higher abundant in NMGs compared to SN_Surr._ tissue in both conditions (Figure 6A), consistent with the results of our previously published study [16]. There was also a slight trend of a higher abundance in CTRL NMGs compared to DLB NMGs (fold change: 1.5), which was consistent with the quantitative comparison, but it did not pass the threshold for significance (adjusted *p*-value: 0.24).

α-synuclein was found to be significantly more abundant in NMGs of DLB cases, while its abundance in all other sample groups was comparable (Figure 6B). While there was no significant difference of α-synuclein abundance in CTRL samples, α-synuclein was found to be 4.2-times higher abundant in DLB NMGs when compared to DLB SN_Surr._ tissue. Additionally, α-synuclein was of significantly higher abundance in NMGs of DLB cases than in NMGs of CTRL.

These results support and validate our global proteome analysis: TH is highly abundant in NMGs of CTRL and DLB cases, while we cannot report a significant decrease in either NMGs or SN_Surr._ tissue between DLB and CTRL cases. For α-synuclein, we can confirm the specific enrichment in NMGs of DLB cases, indicating a potentially disease-related effect.

### 3.7. Proteins Associated with Endosomes, Lysosomes, Ribosomes and Stress Granules Are Comparably Abundant in NMGs of Both Conditions

So far, our analysis provided us with information on proteome-wide differences between NMGs and SN_Surr._ tissue of CTRL and DLB cases. We assume that, while changes in protein abundance may indicate disease-specific changes potentially explaining the role or formation of NMGs under neurodegenerative conditions, comparable protein abundances may hint towards conserved mechanisms present in NMGs regardless of health status.

Therefore, to answer the question of NMG origin, we decided to identify well-known marker proteins associated with endosomes, lysosomes, ribosomes and stress granules, as these structures were previously assumed to be involved in origin, formation or function of NMGs.

To do so, we focused on comparing the protein expression intensities of three reference proteins for each organelle/cellular compartment (Table 3). Differences in protein expression intensities between NMGs of DLB and CTRL cases were rather moderate (ratios between 0.57 and 1.81). Slight trends towards a higher abundance in NMGs of CTRL cases are shown for stress granule marker proteins as well as for ribosomal proteins, which is in line with our quantitative comparison. While endosomal marker proteins are either not changed (EEA1) or tend towards a lower abundance in NMGs of CTRL cases (RAB5C, RAB7A), lysosomal markers show contradictory results with cathepsin D (CTSD) being higher abundant in NMGs of CTRL cases, while lysosome-associated membrane glycoprotein 1 (LAMP1) and lysosome membrane protein 2 (SCARB2) are lower abundant in NMGs of CTRL cases.

To gain a better overview on the presence of ribosomal, lysosomal and endosomal as well as stress granule-associated proteins, we compared our identified proteins in NMGs of CTRL and DLB cases to proteins associated with endosomes (632 proteins), lysosomes (369 proteins) and stress granules (41 proteins) based on the term “subcellular localization” in UniProt [41] as well as a reference list of proteins belonging to either the 40S or 60S ribosomal subunit [43] (79 proteins, Table 4, Supplementary Table S3).

It becomes observable, that the number of proteins identified in NMGs of either CTRL or DLB cases are comparable for all of the four assessed lists. In addition, the overlap between the proteins identified in NMGs of CTRL and DLB cases is very high, reaching 100% for proteins constituting the 80S ribosome as well as proteins associated with stress granules. For endosomes and lysosomes, a similar trend could be observed, as the overlap ranged from 89% to 95%, respectively.

Thus, not only the expression intensities of proteins associated with lysosomes, endosomes, ribosomes and stress granules are comparable in NMGs of DLB and CTRL cases, but also the identified proteins being specific for these four groups are mostly identical. This may hint at a conserved mechanism for NMG generation or for targeting of proteins being transported to NMGs.

## 4. Discussion

Although 0.1% of the population above 65 is affected by DLB [1], pathogenesis remains partially elusive and so far there is no curative treatment available. Since it is assumed that NMGs may be associated with the loss of dopaminergic neurons in the SN [7,23], we set out to compare the proteomic profile of NMGs and SN_Surr._ tissue between DLB and CTRL cases. With this, we aimed to gain insights into disease-specific changes in the SN of DLB patients, either taking place in the NMGs themselves or the surrounding SN tissue. In addition, we focused on proteins with already reported links to formation and function of NMGs to get hints towards potentially conserved mechanisms for NMG formation or proteins relevant for NMG function.

Our complete dataset contains over 2500 identified proteins with sufficient quantitative data for over 1200 of them. Fifty-four proteins were found to be differentially abundant between SN_Surr._ tissue of CTRL and DLB cases, compared to 110 proteins being differentially abundant in NMGs. Based on these results, we can add evidence to several existing hypotheses on NMGs and pathomechanisms of DLB. However, it has to be stated that a comparable study has to be conducted with larger cohort sizes, matched for sex and other characteristics (age, PMI) in the future.

### 4.1. Proteomic Profiling of NMG Surrounding Tissue Points towards Selective Loss of Dopaminergic Neurons, Response to Oxidative Stress and Impaired Protein Degradation

Since we wanted to gain general insights into ongoing neuropathological processes in the SN of DLB cases, we compared the proteomic profile of SN_Surr._ tissue of DLB and CTRL cases with two different approaches. The first approach, based on preselected marker proteins for neurons, dopaminergic neurons, oligodendrocytes and stress granules, provided us with evidence of a specific loss of dopaminergic neurons in DLB patients, observable by the loss of abundance of TH, DDC and DAT in SN_Surr._ samples (see Figure 2). This observation is in line with the regularly reported loss of dopaminergic neurons in DLB [7,8]. However, the comparable abundance of general neuronal markers (SNCA, NEFL, MAPT and APP) in both CTRL and DBL SN tissue is surprising.

In our second approach, we performed a direct comparison of global protein abundances for over 1200 proteins in SN_Surr._ tissue, providing us with promising findings pointing towards ongoing oxidative stress in DLB cases, as GSS was found to be higher abundant in SN_Surr._ tissue of DLB cases, which may indicate a potential response to oxidative stress [44,45,46]. In addition, we found evidence for a potential impairment of protein degradation mechanisms, as UBQLN1, an important regulator of the protein quality control system, was decreased in abundance in SN_Surr._ tissue of DLB cases [47]. Both of these mechanisms, oxidative stress and impaired protein degradation, were previously reported to be common mechanisms in neurodegenerative diseases [48,49], especially in diseases sharing pathological features with DLB such as Alzheimer’s disease and PD. Thus, these mechanisms cannot be seen as disease-specific for DLB.

### 4.2. α-Synuclein and S100A9 Are Highly Enriched in NMGs of DLB Cases, While the Abundance of Ribosomal and Stress Granule Marker Proteins Are Decreased

Since neuromelanin-containing dopaminergic neurons are especially affected by neuropathological changes in DLB, the main focus of the present study was set on the proteomic changes in the NMGs of DLB cases. The general proteomic profiling showed α-synuclein to be of higher abundance in NMGs of DLB cases, which we were able to verify in targeted PRM-experiments, while stress granule marker proteins as well as those for dopaminergic neurons were decreased, with the latter being again in line with the regularly reported selective loss of dopaminergic neurons in the SN. A reduction in the abundance of key stress granule proteins like G3BP1 and TIA-1 was already shown to impair the function of stress granules, thus reducing the cellular ability to respond towards different stress factors [50].

In addition, protein S100A9 was found to be enriched in NMGs of DLB cases, which may hint towards the presence of α-synuclein fibrils, since it was reported that S100A9 is able to modify the fibrilization of α-synuclein and was also found to be present in Lewy bodies in colocalization with α-synuclein [51,52,53,54]. Both findings together may hint towards a potential mechanism behind the selective loss of NMG containing dopaminergic neurons in DLB, namely the formation of neurotoxic α-synuclein fibrils in NMGs, which may be released into the cytoplasm when the NMG membrane is disrupted. However, it must be admitted that while the localization of α-synuclein towards NMGs was already reported to happen in PD [55], the observed increase in abundance may also be a result of the formation of Lewy bodies in the pigmented areas of dopaminergic neurons in the SN.

Another potentially disease-specific mechanism detected in our study was the significant decrease in abundance of several ribosomal and stress granule proteins, observed in the NMGs of DLB cases. Since the presence of functional ribosomes or stress granules still needs further validation, the relevance of this finding cannot be estimated at the moment. However, it is striking that mutations in the EIF4G gene, encoding for a stress granule marker protein which we found to be significantly higher abundant in NMGs of CTRL cases, were already found to be related to PD and DLB [8,56,57]. It was assumed that mutations in the EIF4G1 gene may impair the cellular ability to respond to stress [57,58].

### 4.3. NMGs May Form in a Conserved Mechanism including Endosomal, Lysosomal and Ribosomal Proteins

Since the mechanism of NMG formation as well as their function is still not completely elucidated, we took a closer look at proteins associated with lysosomes, endosomes, ribosomes and stress granules, as all of these cellular compartments were previously discussed as being connected to NMGs [14,15,19]. We found that fold changes of proteins being characteristic for all four compartments remained lower than 2 in NMGs of CTRL and DLB cases (ratios between 0.54 and 1.81) and that the numbers of proteins being associated with these compartments identified in NMGs of both conditions were comparable as well. In addition, the overlap of proteins being present in NMGs of both conditions was remarkably high, ranging from 89% to 100%.

This finding allows for two possible conclusions, namely that (1) proteins identified in NMGs may be specifically transported to NMGs based on a yet unknown mechanism and therefore be relevant for their function or (2) NMGs form from endosomal and lysosomal fusion and include stress granules at any point of this formation process.

## 5. Conclusions

Concluding, we present the first dataset comparing the proteomic profile of NMGs and surrounding SN tissue between CTRL and DLB cases in this exploratory study. Based on this, we stress the selective loss of dopaminergic neurons in the SN of DLB cases, and provide evidence for ongoing oxidative stress and an impairment of the UPS system. In addition, we show that α-synuclein and protein S100A9 are enriched in NMGs of DLB cases, potentially indicating the presence of neurotoxic α-synuclein fibrils. Concerning the ongoing debated research aspects of NMG formation and function, we speculate about a conserved mechanism either assuming the identified proteins associated with lysosomes, endosomes, stress granules and ribosomes to be specifically transported to NMGs and therefore being relevant for their function or being involved in the formation of NMGs.

## Figures and Tables

**Table 1:** Information of study groups.

Group	Sex	Age (In Years)	Ø Age ± SD	PMI (h)	Ø PMI (h) ± SD
Control (n = 5)	Male	91	78.8 ± 12.2	4:00	4:23 ± 2:01
	Male	66		8:00	
	*Female*	*82*		*3:20*	
	*Female*	*89*		*3:20*	
	Female	66		3:20	
Control Validation (n = 2)	*Male*	*79*	*-*	*11:00*	-
	*Male*	*72*		*9:00*	
DLB (n = 5)	Male	87	85.6 ± 1.9	14:00	7:52 ± 5:49
	Female	85		14:20	
	*Female*	*88*		*2:30*	
	*Male*	*83*		*3:15*	
	Female	85		5:20	
DLB Validation (n = 2)	*Female*	*93*	*-*	*1:20*	-
	*Female*	*90*		*3:00*	

Ø Age ± SD: average age and standard deviation; PMI: *post-mortem* interval; Ø PMI (h) ± SD: average *post-mortem* interval and standard deviation. DLB: dementia with Lewy bodies. Mean values and standard deviations were not calculated for cases used for validation. Information on cases included for validation experiments are written in italics.

**Table 2:** Inclusion list for parallel reaction monitoring-experiments.

Protein	Peptide	m/z	Charge State	RT-Window Mean [±1.5 min]
α-synuclein	EGVVHGVATVAEK	432.57	3	24.4
	EGVVHGVATVAEK	648.3513	2	24.3
	EQVTNVGGAVVTGVTAVAQK	643.3531	3	66.8
	EQVTNVGGAVVTGVTAVAQK	964.526	2	66.8
	TVEGAGSIAAATGFVK	739.8961	2	61.0
Tyrosine hydroxylase	AGGPHLEYFVR	415.884	3	43.4
	FDPDLDLDHPGFSDQVYR	712.6605	3	75.6
	TGFQLRPVAGLLSAR	529.3106	3	69.8
Glycerol-3-phosphate-dehydrogenase	NHVVDISESGLITIAGGK	603.995	3	67.3
	LAFLNVQAAEEALPR	821.4516	2	89.5
Cytoplasmic dynein 1 heavy chain 1	ESPEVLLTLDILK	735.4267	2	105.6
	EFGPVVIDYGK	612.319	2	66.1

m/z: mass-to-charge ratio; RT: retention time.

**Table 3:** Abundance levels of marker proteins for endosomes, lysosomes, ribosomes and stress granules in NMGs of CTRL and DLB cases.

Gene Name	Marker for	Mean iBAQ Value CTRL	Mean iBAQ Value DLB	Ratio (CTRL/DLB)
RAB5C	Endosomes	0.000525	0.000561	0.94
RAB7A	Endosomes	0.001026	0.001591	0.64
EEA1	Endosomes	0.0000349	0.0000348	1.00
LAMP1	Lysosomes	0.000256	0.000314	0.82
SCARB2	Lysosomes	0.000500	0.000874	0.57
CTSD	Lysosomes	0.000908	0.000631	1.44
RPS9	Ribosomes	0.004019	0.002987	1.35
RPS7	Ribosomes	0.001508	0.001091	1.38
RPL3	Ribosomes	0.001373	0.001115	1.23
PABPC1	Stress Granules	0.000846	0.000654	1.29
G3BP1	Stress Granules	0.000036	0.000029	1.25
eIF4G1	Stress Granules	0.000097	0.000054	1.81

RAB5: Ras-related protein Rab-5C, RAB7A: Ras-related protein Rab-7a, EEA1: early endosome antigen 1, LAMP1: lysosome-associated membrane glycoprotein 1, SCARB2: lysosome membrane protein 2, CTSD: cathepsin D, RPS9: 40S ribosomal protein S9, RPS7: 40S ribosomal protein S7, RPL3: 60S ribosomal protein L3, PABPC1: polyadenylate-binding protein 1, G3BP1: Ras GTPase-activating protein-binding protein 1, eIF4G1: eukaryotic translation initiation factor 4 gamma 1.

**Table 4:** Summary of identified proteins for endosomes, lysosomes, ribosomes and stress granules in NMGs of CTRL and DLB cases.

List	Number of Included Proteins	Identified in CTRL NMGs (Mean iBAQ > 0)	Identified in DLB NMGs (Mean iBAQ > 0)	Overlap of Proteins Identified in CTRL and DLB NMGs (in %)
Endosome	632	148	140	89
Lysosome	369	97	96	95
Ribosome	79	67	67	100
Stress Granule	41	19	19	100

## Data Availability

The mass spectrometry data have been deposited to the ProteomeXchange Consortium via the PRIDE partner repository with the dataset identifier PXD035310 [34].

## Supplementary Materials

The following supporting information can be downloaded at: https://www.mdpi.com/article/10.3390/cells11223538/s1. Figure S1: Isolation of neuromelanin granules (NMGs) and surrounding *substantia nigra* tissue (SN_Surr._) via LMD, Table S1: Data of protein quantification (LFQ-based), Table S2: Data of protein identification and profile plots (iBAQ-based), Table S3: Comparison of identified proteins with reference protein lists for endosomes, lysosomes, ribosomes and stress granules.
